# Overexpression of SLC25A38 protein on acute lymphoblastic leukemia cells

**DOI:** 10.3892/ol.2014.1947

**Published:** 2014-03-07

**Authors:** HUAYING CHEN, QUANYI LU, YUNWU ZHANG, CUILIN ZHANG, HAN ZHANG, HUAXI XU

**Affiliations:** 1 Department of Hematology, Zhongshan Hospital of Xiamen University, Xiamen, Fujian 361004, P.R. China; 2 Fujian Provincial Key Laboratory of Neurodegenerative Disease and Aging Research, College of Medicine, Xiamen, Fujian 361000, P.R. China; 3 School of Pharmaceutical Sciences, Xiamen University, Xiamen, Fujian 361000, P.R. China

**Keywords:** SLC25A38 protein, molecular target, acute lymphoblastic leukemia

## Abstract

SLC25A38 is a recently identified protein that belongs to the mitochondrial solute carrier family, SLC25. Previous studies have shown that it is a pro-apoptotic protein, which regulates intrinsic caspase-dependent apoptosis. In order to clarify the effect of SLC25A38 protein expression on acute lymphoblastic leukemia (ALL) cells, we detected the expression of SLC25A38 in various cell lines (RPMI 8226, U266, Molt-4 and Jurkat) by western blot analysis. The results indicate that SLC25A38 is highly expressed in the four cell lines. Among 55 leukemia patients (adult, n=32 and infant, n=23), a high expression of SLC25A38 protein was observed in seven infant (7/23, 30.4%) and 15 adult (15/32, 46.9%) ALL patients. Two adult ALL patients that were positive for SLC25A38 were analyzed and the level of SLC25A38 significantly reduced or disappeared following combined chemotherapy, however, reappeared upon ALL recurrence. The expression level was identified to be associated with the proportion of blast cells in the bone marrow. Additionally, SLC25A38 and Notch1 were co-expressed in the four cell lines and the ALL patient samples. The present results show that expression of SLC25A38 is a common feature of ALL cells and may be a novel biomarker for diagnosis, as well as a potential therapeutic target for ALL.

## Introduction

Acute lymphoblastic leukemia (ALL) is a malignancy of hematopoietic stem cell origin with distinctive clinical, immunophenotypic, cytogenetic and molecular biological characteristics. ALL is the most common malignancy in children and has five-year event-free survival rates ranging between 76 and 86% in patients who receive protocol-based therapy. In adults, ALL is a common disease and is generally associated with a worse prognosis. Although intensified chemotherapy protocols may result in 70–90% remission in adult ALL patients, the long-term survival rate is extremely low and the probability of survival is <30–40% (1–9). Consequently, further improvements in the outcome of ALL therapy require the development of novel, targeted and less toxic therapies.

The solute carrier (SLC) family of membrane transport proteins include >300 members organized into >50 families (10). Among these, SLC25 is notable as it is localized on the inner mitochondrial membrane and is, therefore, referred to as a mitochondrial carrier (11). SLC25 is a family of structurally and functionally related proteins that contain six α-helical membrane-spanning regions (12). SLC25 member proteins are encoded by nuclear genes and are synthesized in the cytosol. Newly synthesized proteins subsequently translocate into the inner membranes of mitochondria, where they transport various substrates, such as metabolites, nucleotides and cofactors between the cytoplasm and the mitochondrial matrix (13). SLC25A38 belongs to the SLC25 family (13) and previous studies have found that variations in the SLC25A38 gene, which is located on chromosome 3p22, are responsible for severe pyridoxine-refractory congenital sideroblastic anemia (14–16). The gene encodes a mitochondrial carrier protein required for erythropoiesis and it may act by importing glycine into mitochondria or may act as a transporter of glycine/5-aminolevulinic acid (ALA) across the mitochondrial inner membrane (14–16). Transport of glycine/ALA across the mitochondria is a crucial and rate-limiting step for the synthesis of heme, which is essential in various biological processes, such as respiration, detoxification and signal transduction (17,18). SLC25A38 mutations can lead to glycine/ALA transport disorder, thus affecting the synthesis of heme.

Notch signaling pathway activation is known to contribute to the pathogenesis of a spectrum of human malignancies, including T-cell lymphoblastic leukemia and multiple myeloma (MM) (19–20). The Notch family of proteins is a group of four highly conserved receptors (Notch 1–4) that are expressed on the cell surface and directly regulate gene transcription. It has been reported that Notch1 and its ligand Jagged1 are proteins with important roles in the growth of leukemia cells. High levels of Notch1 and Jagged1 are common in acute myeloid leukemia and chronic lymphocytic leukemia samples from patients, and leukemia cell lines (21). Notch activation is associated with an improved early therapeutic response and increased sensitivity to glucocorticoids (22). Therefore, it is important to investigate the correlation between SLC25 and Notch protein expression in ALL patients.

Recently, our laboratory found that SLC25A38 is a general pro-apoptotic protein that regulates intrinsic caspase-dependent apoptosis by modulating heme biosynthesis. It was demonstrated that SLC25A38 is abundantly expressed in the liver during early embryonic development, thus, indicating the involvement of SLC25A38 in hematopoiesis. In addition, it was found that SLC25A38 was able to form homodimers (23) and an earlier report demonstrated that SLC25A38 is highly expressed in erythroid cells (10). Therefore, we hypothesized that the SLC25A38 protein may be significant in the pathological changes associated with leukemia. To further clarify the significance of SLC25A38 protein expression in ALL, samples were collected from patients with different types of lymphoblastic leukemia, and the abnormal expression of SLC25A38 protein and its impact on the prognosis of ALL was observed.

## Materials and methods

### Cells and antibodies

RPMI 8226 and U266 (the MM cell lines), and Molt-4 and Jurkat (leukemia cell lines) cells were obtained from the Shanghai Cell Bank, Chinese Academy of Sciences (Shanghai, China). All the cell lines were seeded at 0.3×10^6^/ml RPMI-1640 medium, supplemented with 10% fetal bovine serum (FBS) and penicillin/streptomycin, and maintained at 37°C in a humidified atmosphere containing 5% CO_2_.

Rabbit anti-SLC25A38 and anti-Notch1 antibodies were obtained from Abcam (Cambridge, MA, USA), rabbit anti-glyceraldehyde-3-phosphate dehydrogenase (GAPDH) antibodies were obtained from Cell Signaling Technology, Inc. (Beverly, MA, USA) and fluorescence-conjugated secondary antibodies were obtained from Pierce Biotechnology, Inc. (Rockford, IL, USA).

### Patient samples

Fifty-five bone marrow samples were collected at diagnosis from 32 adult patients with ALL (median age, 29 years; range 18–74 years), 23 infant ALL patients (median age, 4 years; range between 10 months and 12 years). Twelve peripheral blood samples were collected from healthy volunteers (median age, 28 years; range, 22–35 years). The patients provided written informed consent for the bone marrow collection for diagnostic and research purposes according to the Declaration of Helsinki and the study was approved by the ethics committee of the Zhongshan Hospital of Xiamen University (Xiamen, China). The leukemia diagnosis was established according to French-American-British (24) and World Health Organization (25) classifications and immunophenotyping was conducted by flow cytometry (equipped with 488 nm and other wavelength excitation lasers; 1×10^6^ cells per tube; BD FACSCalibur Flow Cytometer, BD Biosciences, Franklin Lakes, NJ, USA) using a panel of monoclonal antibodies. Reverse transcription-polymerase chain reaction analysis for BCR-ABL and MLL-AF4 was performed at the time of diagnosis.

### Sample preparation

Bone marrow mononuclear cells were isolated by FICOLL gradient centrifugation with lymphocyte cell separation medium (Tianjin Hao Yang Biological Manufacture Co., Ltd., Tianjin, China) at 350 × g for 30 min at room temperature and cultured in RPMI-1640 containing 10% FBS, l-glutamine and penicillin/streptomycin. The lymphocytes and cell lines were lysed for 30 min on ice in lysis buffer containing freshly prepared protease inhibitors. The cell debris was removed by centrifugation (14,000 × g for 15 min at 4°C). The protein contents of the lysates were determined using the Bio-Rad Protein assay kit (Bio-Rad, Hercules, CA, USA) and a BSA standard.

### Western blot analysis

Equal amounts of protein lysates (40 μg) were analyzed by SDS-PAGE and were electrotransferred to polyvinylidene difluoride membranes. The membranes were washed with 1X Tris-buffered saline 1% Tween-20 (TBST), blocked for 1 h at room temperature (RT) in 5% milk/TBST, and immunoblotted with specific antibodies, which included the rabbit anti-SLC25A38, anti-Notch1 (Abcam) and anti-GAPDH (Cell Signaling Technology, Inc.) monoclonal antibodies. GAPDH served as a loading control. On the subsequent day, the membranes were washed with 1X TBST and incubated with goat anti-rabbit horseradish peroxidase-conjugated secondary antibodies diluted to 1:4,000 in 5% milk/TBST for 1 h at RT. The antibodies were detected using an enhanced chemiluminescence detection kit (Vazyme Biotech Co., Ltd., Nanjing, China). The relative densities of the presenting quantity of target proteins were assessed using Quantity One software (Bio-Rad) followed by normalization against the housekeeping protein, GAPDH.

### Statistical analysis

Data are summarized as means ± standard deviation and comparisons of the means between groups were performed using Student’s t-test. SPSS software for Windows 13.0 was used to determine the P-values and P<0.05 was considered to indicate a statistically significant difference.

## Results

### Overexpression of SLC25A38 protein in leukemia cell lines

**Figure 1:**
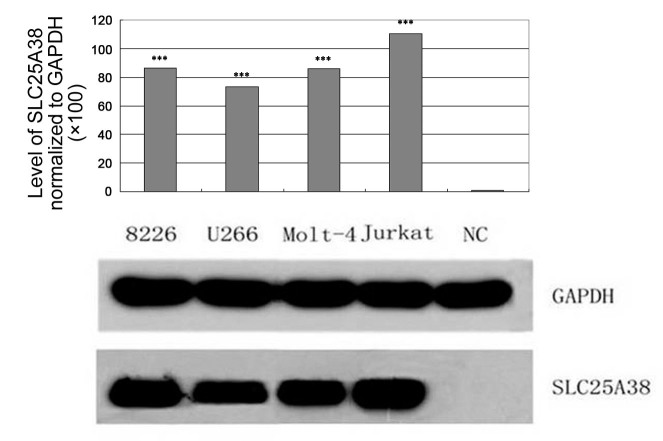
Expression of SLC25A38 protein in the MM and leukemia cell lines. Two MM cell lines (RPMI 8226 and U266 cells) and two leukemia cell lines (Molt-4 and Jurkat cells) were cultured and SLC25A38 protein expression levels were detected using western blot analysis. A significant difference in the SLC25A38 protein expression was identified between the four cell lines and the NC. Data are given relative to the expression of GAPDH. ^***^P<0.0001 compared with the NC. GAPDH, glyceraldehyde-3-phosphate dehydrogenase; MM, multiple myeloma; NC, normal lymphocyte cell control.

Western blot analysis was used to analyze the expression of SLC25A38 protein in various MM and leukemia cell lines. As shown in Fig. 1, an overexpression of SLC25A38 protein was observed in four cell lines. There was no SLC25A38 protein expression detected in the normal lymphocyte cells from the healthy volunteers.

### SLC25A38 protein expression in ALL patients

**Figure 2:**
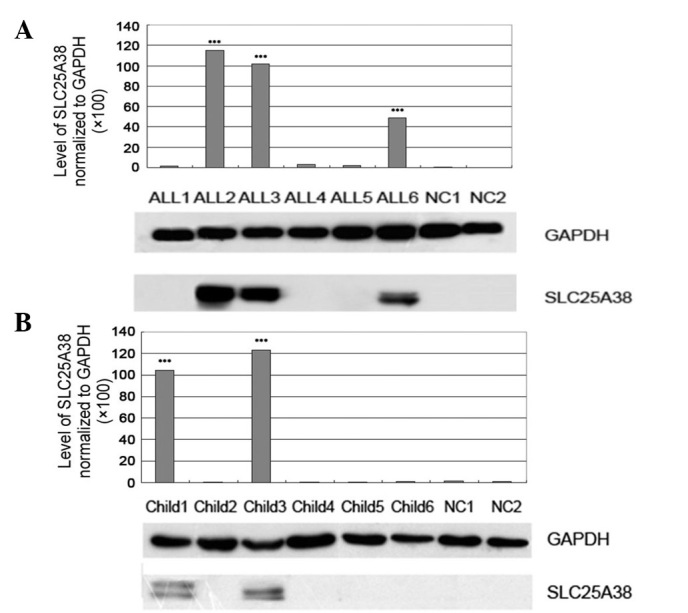
Expression of SLC25A38 protein in ALL patients. Bone marrow samples (n=55) of ALL patients were collected and western blot analysis, using antibodies specific to SLC25A38, was conducted to detect SLC25A38 protein expression. (A) Adult ALL patient samples. (B) Infant ALL patient samples. A significant difference was identified between the ALL patient samples, and NC1 and NC2. Data are given relative to the expression of GAPDH. ^***^P<0.0001 compared with NC1 and NC2. GAPDH, glyceraldehyde-3-phosphate dehydrogenase; ALL, acute lymphoblastic leukemia; NC1/2, normal lymphocyte cell control 1/2.

The expression of SLC25A38 protein was detected in the 55 leukemia patients and the results showed that a high expression of SLC25A38 was common in adult (15/32, 46.9%) and infant (7/23, 30.4%) ALL patients (Fig. 2A and B).

### Expression level of SLC25A38 protein reflects the tumor burden

**Figure 3:**
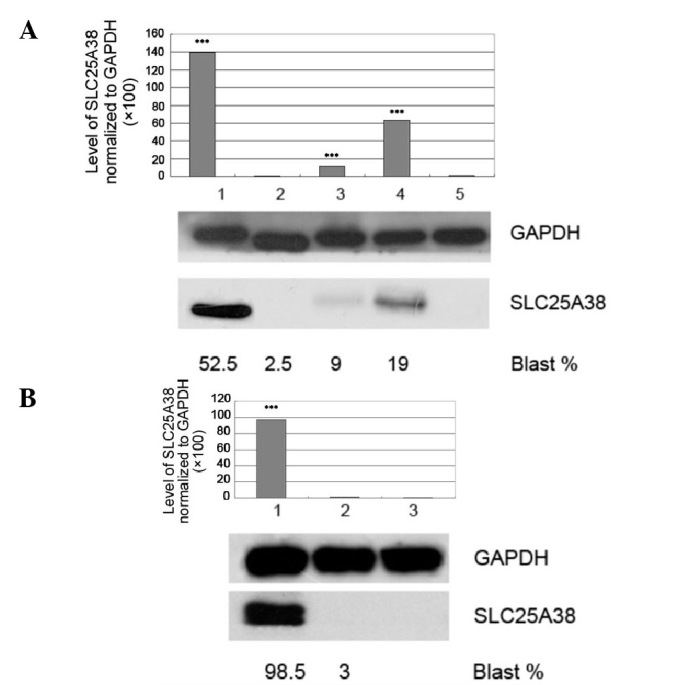
Expression of SLC25A38 protein was associated with the blast cell burden. Two adult ALL patients with SLC25A38-protein positive expression were observed. Bone marrow mononuclear cells were collected from the patients at different phases (including newly diagnosed, remission and relapse). The results showed that the level of SLC25A38 protein expression was associated with the blast cell proportion in the bone marrow. During complete remission, SLC25A38 protein expression ceased, however, it was observed during ALL relapse. (A) The level of SLC25A38 protein expression with varying blast cell proportions in the bone marrow: Lanes 1–4, adult ALL patient no. 1; lane 5, NC. (B) The level of SLC25A38 protein expression with varying blast cell proportions in the bone marrow: Lanes 1–2, adult ALL patient no. 2; lane 3, NC. Data are presented relative to the expression of GAPDH. ^***^P<0.0001 compared with the NC. GAPDH, glyceraldehyde-3-phosphate dehydrogenase; ALL, acute lymphoblastic leukemia; NC, normal control.

To obtain a more reliable conclusion, two adult ALL patients with positive SLC25A38 were observed. It was found that the SLC25A38 expression level significantly reduced or disappeared after receiving combined chemotherapy, however, the expression returned on ALL recurrence (Fig. 3A and B). The results also indicated that the SLC25A38 expression level was associated with the proportion of blast cells within the bone marrow.

### Notch1 protein expression on SLC25A38-positive cells

**Figure 4:**
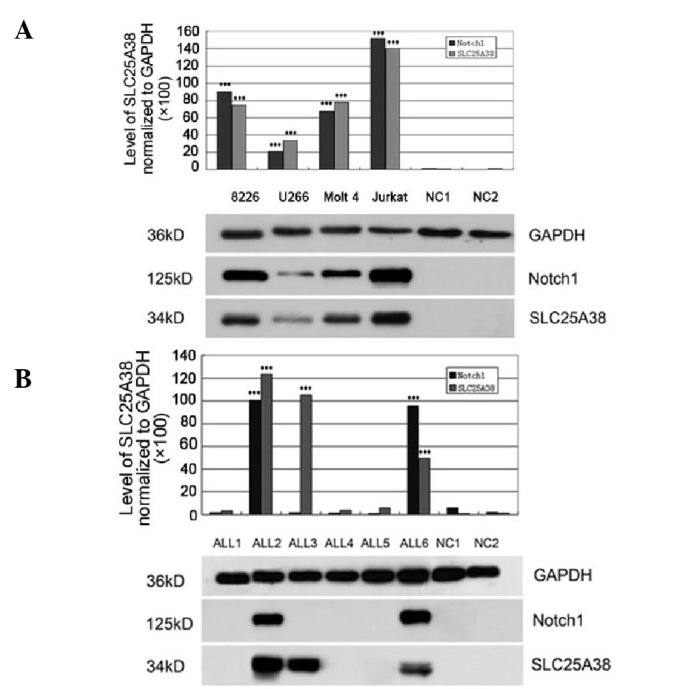
Notch1 protein expression on SLC25A38-protein positive cells. Notch1 protein expression was detected in the cell lines as well as the ALL patient samples. The results demonstrated that SLC25A38 and Notch1 proteins were co-expressed in the four cell lines and the ALL patient samples. (A) SLC25A38 and Notch1 protein expression in four different cell lines. (B) SLC25A38 and Notch1 protein expression in ALL patient samples. Data are given relative to the expression of GAPDH. ^***^P<0.0001 compared with NC1 and NC2. GAPDH, glyceraldehyde-3-phosphate dehydrogenase; ALL, acute lymphoblastic leukemia; NC1/2, normal lymphocyte cell control 1/2.

In the present study, Notch1 protein expression was analyzed in the cell lines and the patient samples. As shown in Fig. 4A, U266 cells marginally expressed the Notch1 protein; RPMI 8226, Molt-4 and Jurkat cells expressed Notch1, with the highest levels observed in the Jurkat cells. In addition, Notch1 and SLC25A38 proteins were co-expressed in the cell lines. Notch1 overexpression was detected in more than half of the adult ALL patients who were SLC25A38-positive (8/15 ALL patients, 53.3%); however, Notch1 protein expression was not detected in the patients with negative SLC25A38 protein expression (Fig. 4B).

## Discussion

ALL is a genetically heterogeneous disease in which different genetic alterations cooperate to promote the uncontrolled clonal proliferation and survival of leukemic lymphoblasts (26–29). The outcome of ALL patients has improved significantly over the last two decades as a result of combination chemotherapy. However, patients with several ALL subtypes continue to show a poor prognosis, and treatments are responsible for the short and long-term toxicities experienced by certain long-term surviving patients. Unfortunately, the majority of patients eventually experience relapse or succumb to their disease after developing drug resistance (30). However, monoclonal antibodies, gene inhibitors and upregulation of microRNAs (31–33) may provide promising tools in the search for ALL-targeted therapy.

As it was discovered recently, the normal physiological function of SLC25A38 protein remains obscure. Similar to the observations of the present study, a study by Guernsey *et al* (14) indicates that the SLC25A38 protein is related to heme synthesis and oxygen transportation in cells. Downregulation of SLC25A38 resulted in a significant decrease in the levels of cellular heme, which affects mitochondrial respiration and oxidative phosphorylation. To the best of our knowledge, this is the first study to report SLC25A38 protein expression in ALL and preliminarily identify that a high expression of SLC25A38 is a common phenomenon in ALL, with certain clinical significance. In cellular experiments, it was found that four cell lines expressed high levels of SLC25A38. Additionally, SLC25A38 expression was observed to be common in adult ALL patients (15/32, 46.9%) and in infant ALL patients (7/23, 30.4%).

Notch signaling affects multiple processes that govern normal morphogenesis, programmed cell death and cellular proliferation. Altered Notch signaling has been associated with various malignancies, including pancreatic, breast, leukemia and lymphoma (34). Activating mutations in the Notch1 gene are present in >50% of human T cell-ALL (T-ALL) cases making Notch1 the most prominent oncogene, which is specifically involved in the pathogenesis of this disease, and defining T-ALL as a disease that is primarily characterized by aberrant Notch1 activation (35–38). In the present study, the expression of the SLC25A38 and Notch1 proteins was identified to be common in cell lines as well as in the majority of ALL patients exhibiting positive SLC25A38 expression. Therefore, it was hypothesized that the overexpression of SLC25A38 may be connected to the activation of the Notch signaling pathway.

In conclusion, in the present study the overexpression of SLC25A38 protein in the cell lines and in ALL patients was observed. The data indicates that future studies are required to determine the role of SLC25A38 in the pathogenesis of leukemia, and establish whether overexpression of this protein regulates the proliferation, differentiation and apoptosis of leukemia cells. The present findings are considered to be important for determining novel leukemia markers and molecular targets for the treatment of leukemia.
